# Comparison of the Response to Pulpal Sensibility Tests in Well-Controlled and Uncontrolled Type II Diabetes Mellitus Patients: A Cross-Sectional Study

**DOI:** 10.1155/2022/6197070

**Published:** 2022-09-13

**Authors:** Fatemeh Owlia, Faezeh Zarezadeh, Sara Jambarsang, Maryam Kazemipoor

**Affiliations:** ^1^Department of Oral and Maxillofacial Medicine, School of Dentistry, Shahid Sadoughi University of Medical Sciences, Yazd, Iran; ^2^Department of Endodontics, School of Dentistry, Shahid Sadoughi University of Medical Sciences, Yazd, Iran; ^3^Department of Bio-Statistics and Epidemiology, Shahid Sadoughi University of Medical Sciences, Yazd, Iran

## Abstract

**Introduction:**

Diabetes mellitus is a metabolic disorder in which impairment of sensory fibers would be anticipated. The present study would assess the dental pulp response to cold and EPT sensibility tests in patients with type 2 diabetes, both well-controlled and uncontrolled.

**Materials and Methods:**

One hundred two maxillary central incisors, belonging to participants aged 35–67 years, were included in this survey. At last, 51 diabetic patients were allocated to each group of well-controlled (HbA1C < 7) and uncontrolled (HbA1C ≥ 7). Electric and cold pulpal vitality tests were conducted for all teeth. Statistical analysis was performed with Student's *t*-test, the chi-square test, and the multiple linear regression model. A *P* value less than 0.05 was considered significant.

**Results:**

Based on the results of this study, the mean value of response to EPT was 4.51 ± 2.06 and 4.41 ± 1.85 in well-controlled and uncontrolled diabetic patients, respectively. Also, the pulpal responses to cold and EPT tests had no significant differences between the two groups (*P* > 0.05).

**Conclusion:**

Tooth responses to the cold and EPT sensibility tests were not different in well-controlled and uncontrolled diabetic patients. Despite no statistically significant correlation, male diabetic patients in the uncontrolled group showed a lower sensory response threshold to EPT compared to a well-controlled group.

## 1. Introduction

Diabetes mellitus is the most common metabolic disorder in the world. Epidemiologic studies revealed that the prevalence of diabetes mellitus in 2019 was 9.3% and was rising to 10.2% by 2030 and 10.9% by 2045. The prevalence of diabetes mellitus (DM) has risen rapidly during the past decades in Iran and other low- and middle-income countries [1]. It is estimated that in the year 2030, nearly 9.2 million Iranians are likely to have diabetes [1].

Diabetes mellitus is a metabolic disorder in which impairment of insulin secretion, defective insulin action, or both lead to hyperglycemia [2, 3]. Some of the signs and symptoms of type 1 and type 2 diabetes include increased thirst, frequent urination, extreme hunger, unexplained weight loss, presence of ketones in the urine, fatigue, irritability, blurred vision, slow-healing sores, and frequent infections, such as gum or skin infections [4, 5].

Oral manifestations of uncontrolled diabetes can be summarized in xerostomia, a burning mouth sensation related to neuropathy, impaired/delayed wound healing, heightened incidence and severity of infections, secondary infection with candidiasis, parotid salivary gland enlargement, gingivitis, and/or periodontitis [6].

Nervous and vascular systems are the two that are primarily affected by the complications of diabetes [7]. These complications are generally divided into two categories: microvascular and macrovascular, which could affect various organs of the body such as the eyes, nerves, kidneys, blood vessels, and immune system [8]. Lack of blood sugar control is associated with an elevated risk of vascular complications, such as heart attack, stroke, and neuropathy [9], as well as depression [10]. It has been reported that 20% of patients with type 2 diabetes have diabetic neuropathy [11].

Sensibility pulpal tests (thermal and electrical) have been used to indirectly assess the status of dental pulp nerve fibers. Sensibility tests, although subjective and patient-related, are useful in assessing pulpal vitality [12]. The electrical pulp tester (EPT) stimulates the healthy Að nerve fibers in the pulp-dentin complex by applying an electric current to the tooth surface [13]. The positive response is due to an ionic change in the fluid inside the dentinal tubules, which engenders local depolarization and thus produces the action potential in healthy and intact A delta nerve fibers [14]. EPT is particularly useful in teeth with limited fluid flow through dentinal tubules, such as with dentinal sclerosis [15]. The dental pulp nerve fibers can be affected by neuropathic changes in different conditions [16, 17]. Thermal sensibility tests induce tubular fluid to move, followed by irritating the nerve fiber endings located inside the dentinal tubules, which act as mechanoreceptors [18]. Materials available for cold tests include dry ice (CO_2_), ice, and refrigerant sprays such as tetrafluoroethane, butane, propane, isobutene, dichlorofluoromethane (DDM), and ethyl chloride [12]. Research has shown that the accuracy of CO_2_ and refrigerants to determine the sensibility of the pulpal nerve fibers is superior to the electrical test pulp [19].

Diabetic neuropathy affects A delta nerve fibers [20], and therefore, the response of pulpal nerves to sensibility tests could be hampered in diabetic patients. The aim of the present study was to compare the response of the dental pulp nerve to pulpal sensibility tests (cold and electrical) in patients with type 2 well-controlled and uncontrolled diabetes.

## 2. Materials and Methods

### 2.1. Study Population

All the diabetic patients were evaluated by the investigator; if they met the inclusion criteria, they were enrolled in the study. One hundred two type 2 diabetic patients have participated in this descriptive cross-sectional study. The patients were selected among the diabetic patients of the Yazd diabetes center. At last, 51 patients with a recent HbA1C ≥ 7 as an uncontrolled group and 51 patients with a recent HbA1C < 7 as a well-controlled group enrolled in this study.

### 2.2. Ethical Consideration

All the experimental procedures in the present study were approved by the Ethics Committee of Research Shahid Sadoughi University of Medical Sciences, Yazd (IR.SSU.REC. 1399.046). After the research method was explained to the participants, all of them signed an informed consent form.

### 2.3. Inclusion and Exclusion Criteria

Inclusion criteria for the participants were 35- to 67-year-old patients with type 2 diabetes according to the American Diabetes Association criteria. Patients with a history of taking tricyclic antidepressants, anticonvulsants, and antihypertensive medication during the last 3 months, any systemic diseases other than diabetes, and the use of different amounts and types of analgesics during the 48 hours before the sensibility tests were excluded from the present study. Both groups were matched based on age and gender.

Local factors composed of extensive filling, dental caries, a history of trauma or orthodontic treatment, and periodontal problems were also considered [21]. The pulpal sensibility tests were conducted to the maxillary intact central teeth without any restoration, caries lesion, periodontal problem, sensitivity to percussion, history of trauma, and orthodontic treatment.

## 3. Methods

Before testing, the surface of the teeth was made free of debris, calculus, and plaque. Teeth were first dried and isolated with a cotton roll, and an electrocardiography gel (BP Ultra Gel, Turkuaz Saglik Co., Turkey) was applied on the buccal face of the crown as an interface media. An electric pulp tester (EPT) (Gentle-pulse, Parkell, USA) probe was placed on the sound coronal third of the labial surface, and the “tingling” sensation felt by the patient once the increasing voltage reached the pain threshold was recorded.

Cold testing with ethyl chloride was accomplished by using a large cotton pellet on the buccal surface of the tooth for 15 seconds, or until the patient indicated a response. For each, the cold and the electrical stimulation threshold of the central maxillary incisor were measured and recorded. The patient response to the cold test and the time interval between the cold application and patient response were also recorded.

### 3.1. Statistical Analysis

SPSS software (version 22, IBM Corporation, Armonk, NY) was used for analysis of the data. Results were expressed as a mean and standard deviation. Inferential statistics were applied with the use of the Student's *t*-test and the chi-square test. A multiple linear regression model was used to adjust the relationship of test responses for confounders (*P* < 0.05—statistically significant at a 95% confidence interval).

## 4. Results

### 4.1. Sociodemographic Data

One hundred two diabetic patients fall into 2 groups: well-controlled (*n* = 51) and uncontrolled (*n* = 51). The mean age of the studied subjects was 51, ranging from 35 to 67. In this regard, 24 individuals were male (24.6%) and 78 subjects were female (76.4%). In both groups, the percent of patients with neuropathy was higher than that of patients without neuropathy. Mentioning this point, the number of involved patients in the uncontrolled group was higher (39.2% in return to 15.7%). The demographic characteristics and pulp responses of the two groups are set out in Table 1.

### 4.2. Pulp Sensibility Results

Overall, the results presented below indicate that there is no significant difference between the two groups in pulpal responses to sensibility tests. The mean ± SD responses (EPT and cold test) for well-controlled and uncontrolled diabetic patients are presented in Table 1. Adjustment for age, sex, and duration of diabetes did not change the results materially (Table 2).

**Figure 1:**
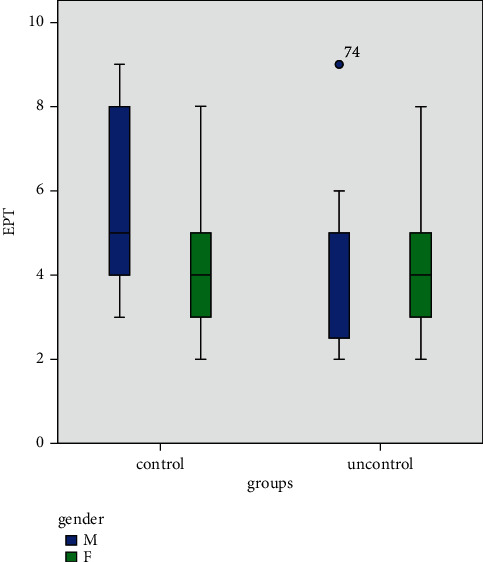
The relationship between EPT and diabetes based on gender.

In spite of no statistically significant correlation, male diabetic patients in the uncontrolled group displayed a lower sensory response threshold to EPT in comparison with the well-controlled group (Figure 1).

**Figure 2:**
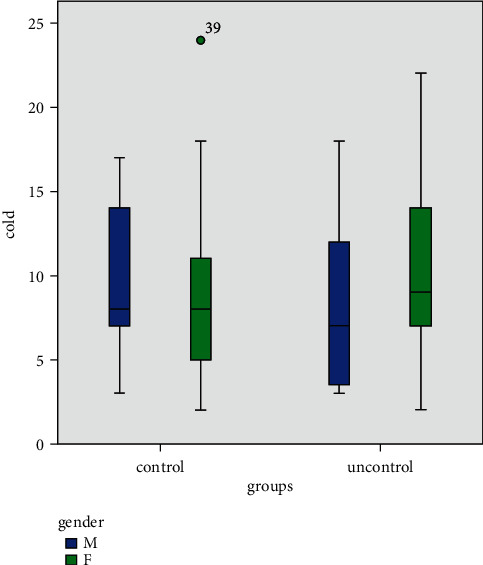
The relationship between the cold test and diabetes based on gender.

It can be seen from Figure 2, that the response threshold to a cold test in male participants of the uncontrolled diabetic group was lower than that in the well-controlled group, but in females, this result was reversed.

**Figure 3:**
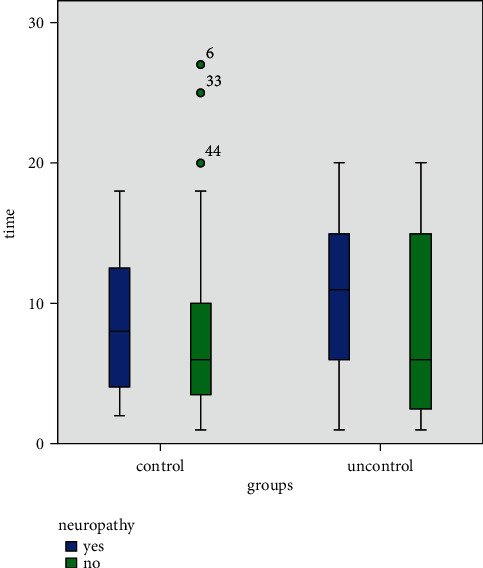
Relationship between duration of diabetes and neuropathy based on gender.

Diabetic patients with more than a 5-year history of diabetes had a significantly higher rate of neuropathy (*P*=0.01). According to the results, patients with neuropathy had a longer duration of diabetes in both groups (Figure 3).

## 5. Discussion

There was no significant difference between the well-controlled and the uncontrolled diabetic groups regarding pulpal responses.

The sample size in the present survey was larger than in some literature [10, 22, 23] and similar to the study by Tavakolinejad Kermani et al. [24]. Since various factors, including the health status of the patients, could affect the response to sensibility tests, strict inclusion criteria were applied in the present study [22]. Patients with systemic diseases other than diabetes did not participate in this study. Patients were not entered into the evaluation process, if they had a history of taking tricyclic antidepressants, anticonvulsants, and antihypertensive medication during the last 3 months [25]. Systemic doses of different types of analgesics for 48 hours could also alter the EPT records. Tooth-related interfering factors, including extensive fillings, dental caries, a history of trauma, and periodontal problems, which induce bias in the results of the EPT test [26–28] are also controlled in the present study.

Aging has a negative impact on the results of the EPT [24, 29]. Therefore, the mean age of participants in both groups who participated in the present survey was similar, with no significant difference. Based on multiple linear regression analysis, aging had a significant positive relation regarding response time to the cold test. It means that the sensory response threshold to cold is enhanced with aging. It could be explained by the deposit of secondary dentin and limited fluid movement in dentinal tubules of aged dentin [30–32]. In a recent study, there was a significant correlation between aging and a reduction in the sensibility of maxillary premolars in diabetic patients to the cold test [24].

Due to more accessibility, easier isolation, lower caries, and point connections to adjacent teeth, the anterior teeth were a more suitable candidate for the sensibility tests. The central maxillary incisor was selected by virtue of central teeth having a lower threshold to EPT than other anterior teeth [26, 33]. The direct pathway of the dentinal tube, the higher concentration of neural components, the low enamel thickness and the low voltage required to stimulus led to the selection of one-third of the incisal edge of teeth for the sensibility pulp tests [34].

Diabetes could impact oral tissues, leading to the expression of inflammatory mediators and modifications of the structural components of dental pulp [35]. Also, diabetes may complicate blood supply in the dental pulp, which could indirectly affect the response of pulpal sensory fibers.

Group allocation in this study was similar to that reported in the literature [22, 24]. In some studies, diabetic patients were compared with healthy individuals [24, 36]. In a similar study [24], the cut-off point of HbA1C in diabetic patients was considered 10%, but we considered 7% to allocate participants regarding the American Diabetes Association guideline in 2021 [37].

In this survey, standard deviations of response threshold to EPT were different between the two genders. It may be related to the unequal distribution of two genders in two groups. The reason for the unequal distribution and the greater number of women was related to the lack of intention to provide regular follow-up for male patients. The presence of diabetic microangiopathy and the effects of female hormones on pulpal sensitivity testing could be the other possible reasons [38].

In some studies, the neuropathy evaluation in diabetic patients was carried out on the peripheral limbs [36]. To date, no study has been found about facial neuropathy based on glycemic control in diabetic patients. Considering the lack of studies in this regard, this survey was designed as a first step for designing future experiments. In this study, self-reported neuropathy was documented by asking questions about tingling sensations or other symptoms related to neuropathy or taking medications to relieve similar symptoms, like gabapentin. In the previous literature, some indices like nerve conduction and diabetic polyneuropathy were applied [10, 20]. Patients with a higher percentage of HbA1C may not respond to the sensibility pulp test accurately. Diabetic neuropathy could influence on Að fibers [20]. Fenn et al. [7] dedicated neuropathy sensory impairment was associated with HbA1C, and duration of diabetes. Despite the partial difference, there was no significant difference in response to EPT and the cold test between the two groups. Although HbA1C is an independent risk factor for peripheral neuropathy [39], Lv et al. demonstrated diabetic patients with peripheral neuropathy had higher rate of glycated HbA1C [22]. It sounds that wide biochemical differences in uncontrolled diabetic patients would be mattered. This study found that diabetic patients with more than a 5-year history of diabetes, had a significantly high rate of neuropathy. This finding goes in line with Nisar et al.'s [10] study. They pondered a cut-off point of 3 years for comparison. The correlation between the duration of diabetes and neuropathy is confirmed by the literature. It may be related to more exposure to risk factors, late diagnosis of neuropathy, or poor control of metabolic disorders [40–42].

Changes in EPT responses could be interpreted as a change in pulpal nerve conduction. It should be analyzed along with the results of pulpal sensibility tests. It does not disclose any histologic data about pulp conditions [26]. A study revealed that the current perception thresholds of the bilateral median nerve and sural nerve were significantly lower in the diabetic group [22]. Kazemipoor et al. found that the dedicated tooth response to the EPT sensibility test may alter in anemic patients [43]. Also, a study mentioned that Að and C fibers pain threshold values were higher in diabetic patients than in the control group. Having no myelin in C fibers, attributed to damage occurring more easily and earlier in diabetic patients. Kukidomehe et al demonstrated that diabetic patients with neuropathy had significantly higher Að, and C fiber pain threshold values than patients without neuropathy [20]. Að fibers are involved in EPT, but they may be sensitized and would respond faster than usual even in the early stage of diabetes. Kazemipoor and Mahmoodi revealed that, after pulpotomy and removal of coronally pulpal tissue, in the absence of myelinated fibers, the tooth response to the EPT sensibility test was also observed. In this regard, both myelinated Að and unmyelinated C fibers could respond to the EPT test [44].

In a recent study, there was no statistically significant correlation between the sensibility pulp tests and diabetes [24]. In spite of no significant relationship, the cold test results were lower in male diabetic patients of the uncontrolled group.

The current study must be considered in light of certain limitations. On the basis of the method, the rate of subjective neuropathy in this survey was recorded by self-declaration. The small sample size in the current study suggests caution in interpreting the results. A similar study in diabetic patients with higher HbA1C showed neuropathy remains an area in need of future research. This study is important and novel as a first step for designing the next experiments. There is clearly much room for further research in this regard.

## 6. Conclusion

Based on the results of the present study, tooth responses to the cold and EPT tests have not shown statistically significant differences in well-controlled and uncontrolled diabetic patients. In spite of no statistically significant correlation, male diabetic patients in the uncontrolled group revealed a lower sensory response threshold to EPT in comparison with the well-controlled group.

## Figures and Tables

**Table 1:** Comparison of demographic characteristics and pulp responses of two groups with well-controlled and uncontrolled diabetes.

	Well-controlled diabetes (*N* = 51)	Uncontrolled diabetes (*N* = 51)	*P* value
*Demographic*			
Age	52.22 ± 7.67	50.12 ± 6.86	0.149^*∗*^
HbA1c	6.44 ± 0.61	8.83 ± 1.27	<0.001^*∗∗*^
Duration of diabetes (year)	7.90 ± 6.12	9.22 ± 5.99	0.276^*∗*^
Gender			0.408^*∗∗*^
Female (%)	34 (74.5)	40 (78.4)	
Male (%)	13 (54.2)	11 (45.8)	
Neuropathy			0.007^*∗∗*^
Yes (%)	8 (15.7)	20 (39.2)	
No (%)	43 (84.3)	31 (60.8)	

*Response*			
EPT	4.51 ± 2.06	4.41 ± 1.85	0.801^*∗*^
Cold	8.65 ± 4.59	9.65 ± 4.69	0.280^*∗*^

Data are presented as mean ± SD, frequency, and percent. ^*∗*^*T*-test. ^*∗∗*^Chi-square.

**Table 2:** Comparison of EPT and cold tests between well-controlled and uncontrolled diabetes groups based on a regression model.

Response	Regression coefficient	95% CI	*P* value	*R* squared
EPT				
Model I^†^	−0.098	(−0.869, 0.673)	0.801	0.001
Model II^‡^	−0.040	(−0.817, 0.736)	0.918	0.030
Model III^§^	0.002	(−0.792, 0.795)	0.997	0.033
Cold				
Model I	1.000	(−0.826, 2.860)	0.280	0.012
Model II	1.311	(−0.502, 3.124)	0.154	0.067
Model III	1.437	(−0.413, 3.288)	0.126	0.072

^*∗*^CI, confidence interval. ^†^Not adjusted. ^‡^Adjusted for age and sex. ^§^Adjusted for all variables included in model II and duration of diabetes.

## Data Availability

All data analyzed during this study are included in this published article. Additional data/files may be obtained from the corresponding author upon reasonable request.
